# Feasibility study of adaptive radiotherapy with Ethos for breast cancer

**DOI:** 10.3389/fonc.2023.1274082

**Published:** 2023-11-13

**Authors:** Arthur Galand, Jessica Prunaretty, Nicolas Mir, Aurélie Morel, Céline Bourgier, Norbert Aillères, David Azria, Pascal Fenoglietto

**Affiliations:** Radiotherapy Department, Montpellier Regional Cancer Institute, Montpellier, France

**Keywords:** breast cancer, adaptive radiotherapy, AI, CBCT, ethos

## Abstract

**Purpose:**

The aim of this study was to assess the feasibility of online adaptive radiotherapy with Ethos for breast cancer.

**Materials and methods:**

This retrospective study included 20 breast cancer patients previously treated with TrueBeam. All had undergone breast surgery for different indications (right/left, lumpectomy/mastectomy) and were evenly divided between these four cases, with five extended cone beam computed tomography (CBCT) scans per patient. The dataset was used in an Ethos emulator to test the full adaptive workflow. The contours generated by artificial intelligence (AI) for the influencers (left and right breasts and lungs, heart) and elastic or rigid propagation for the target volumes (internal mammary chain (IMC) and clavicular lymph nodes (CLNs)) were compared to the initial contours delineated by the physician using two metrics: Dice similarity coefficient (DICE) and Hausdorff 95% distance (HD95). The repeatability of influencer generation was investigated. The times taken by the emulator to generate contours, optimize plans, and calculate doses were recorded. The quality of the scheduled and adapted plans generated by Ethos was assessed using planning target volume (PTV) coverage, homogeneity indices (HIs), and doses to organs at risk (OARs) via dose–volume histogram (DVH) metrics. Quality assurance (QA) of the treatment plans was performed using an independent portal dosimetry tool (EpiQA) and gamma index.

**Results:**

On average, the DICE for the influencers was greater than 0.9. Contours resulting from rigid propagation had a higher DICE and a lower HD95 than those resulting from elastic deformation but remained below the values obtained for the influencers: DICE values were 0.79 ± 0.11 and 0.46 ± 0.17 for the CLN and IMC, respectively. Regarding the repeatability of the influencer segmentation, the DICE was close to 1, and the mean HD95 was strictly less than 0.15 mm. The mean time was 73 ± 4 s for contour generation per AI and 80 ± 9 s for propagations. The average time was 53 ± 3 s for dose calculation and 125 ± 9 s for plan optimization. A dosimetric comparison of scheduled and adapted plans showed a significant difference in PTV coverage: dose received by 95% of the volume (D95%) values were higher and closer to the prescribed doses for adapted plans. Doses to organs at risk were similar. The average gamma index for quality assurance of adapted plans was 99.93 ± 0.38 for a 3%/3mm criterion.

**Conclusion:**

This study comprehensively evaluated the Ethos^®^ adaptive workflow for breast cancer and its potential technical limitations. Although the results demonstrated the high accuracy of AI segmentation and the superiority of adapted plans in terms of target volume coverage, a medical assessment is still required.

## Introduction

Adaptive radiotherapy (ART) takes into account the patient’s anatomical and physiological variations to optimize treatment (1). Since its conceptualization by D. Yan et al. (2), ART has undergone several developments that have allowed it to become an increasingly studied radiotherapy technique (3, 4), already clinically applied to numerous sites such as the lung, bladder, prostate, and cervix (5). There are two categories of ART: offline and online.

Offline ART is defined as the adjustment of the treatment plan between delivered fractions. It may consist of redefining the clinical target volume (CTV) and planning target volume (PTV) based on images from the first sessions (offline composite) (6–8) or creating a treatment plan based on a library of personalized or non-personalized images (individualized or non-individualized plan of the day) (7, 9).

Following the emergence of artificial intelligence (AI), online adaptive radiotherapy (oART) is attracting increasing interest in the clinical field. It allows a complete real-time workflow to be implemented while the patient is on the treatment couch. During the on-couch session, the image of the day is acquired, enabling a deformation matrix to be obtained by deforming the simulation image with the image of the day. Subsequently, contours are generated by AI or propagated via rigid or deformable image registration (DIR). These contours can then be used to create a treatment plan in real time (10, 11).

A number of devices are currently available for oART treatment: MRIdian (ViewRay Inc., Cleveland, OH, USA) and Unity (Elekta AB, Stockholm, Sweden) perform magnetic resonance imaging, while Ethos (Varian Medical Systems, Palo Alto, CA, USA) performs cone beam computed tomography scans (12). oART is already being used for certain pelvic cancers, particularly prostate cancer (13, 14), as in the case of the Montpellier Regional Cancer Institute (ICM), where two Ethos machines are used to perform oART with artificial intelligence for patients with pelvic cancers. Adaptive radiotherapy for breast cancer using Ethos has been previously investigated for partial breast irradiation (15, 16). However, to our knowledge, this technique has not yet been used for breast cancer including regional lymph nodes. Nevertheless, for breast cancer patients, many anatomical variations exist: heart movement, breathing, and arm position can all lead to changes in breast position and shape, as can seroma and swelling following radiation or surgery. The oART would therefore allow treatment margins to be reduced and, consequently, the volume irradiated (17).

The aim of this study is therefore to assess the feasibility of oART in breast cancer patients treated with Ethos in preparation for the upcoming initiation of a clinical trial to evaluate the clinical outcomes of adaptive radiotherapy with Ethos in radiosensitive breast cancer patients.

## Materials and methods

### Patient selection

Data from 20 patients treated for invasive breast carcinoma between November 2021 and December 2022 were retrospectively selected. Patients were included regardless of age, histology, tumor grade, surgical treatment (lumpectomy or mastectomy), or neoadjuvant chemotherapy. The 20 patients were stratified into four groups: right breast, left breast, right chest wall, and left chest wall. Patient characteristics are described in **Table 1**.

**Table 1:** Patient characteristics, including treatment side, type of surgery, and volume of breast/chest wall CTV.

Patient	Laterality	Type	CTV breast/chest wall volume (cc)
1	Right	Conserving surgery	802.5
2	Right	Conserving surgery	489.7
3	Right	Conserving surgery	904.6
4	Right	Conserving surgery	501.2
5	Right	Conserving surgery	381.5
6	Left	Conserving surgery	575.0
7	Left	Conserving surgery	754.2
8	Left	Conserving surgery	692.5
9	Left	Conserving surgery	870.3
10	Left	Conserving surgery	475.4
11	Right	Mastectomy	415.9
12	Right	Mastectomy	274.0
13	Right	Mastectomy	527.8
14	Right	Mastectomy	250.3
15	Right	Mastectomy	516.1
16	Left	Mastectomy	384.5
17	Left	Mastectomy	237.6
18	Left	Mastectomy	368.5
19	Left	Mastectomy	621.1
20	Left	Mastectomy	623.6

CTV, clinical target volume.

### Treatment planning

All patients were previously treated with TrueBeam in volumetric-modulated arc therapy (VMAT) irradiation. Patients underwent computed tomography (CT) imaging (GE Optima CT580, General Electric Healthcare, Waukesha, WI, USA) with a 2.5-mm slice thickness in a supine position during free breathing and with both arms over the head with personalized foam cushions. In this study, we chose to replan the simulated treatment using an intensity-modulated radiotherapy (IMRT) beam geometry rather than VMAT because of the longer optimization and calculation times with VMAT, which we considered unacceptable for daily adaptive sessions. For this reason, IMRT plans with 13 fields and 6-MV flattening filter-free (FFF) beams were designed for each patient using the Eclipse treatment planning system (v15.6, Varian Medical Systems, Palo Alto, CA, USA) and imported into the Ethos^®^ solution. Prescriptions for target volumes were 52.2 Gy for the tumor bed (boost) and 42.3 Gy for the breast, the internal mammary chain (IMC), and the clavicular lymph nodes (CLNs) in 18 fractions. CTV–PTV margins were set at 2 mm for all locations except the IMC, for which the margin is 5 mm. The dose constraints for the CTVs and the organs at risk (OARs) are shown in **Table 2**.

**Table 2:** Dose constraints for the CTVs and the organs at risk.

CTV constraints	
CTV Boost	D_95%_ ≥ 49.6 Gy
CTV Breast/Chestwall	D_95%_ ≥ 40.2 Gy
CTV nodes (IMC and CLN)	D_95%_ ≥ 40.2 Gy
OAR constraints	
Heart	V_17Gy_ < 10%
Ipsilateral lung	V_17Gy_ < 30%
Lungs	V_17Gy_ < 22%
Brachial plexus	D_max_ < 46.25 Gy
Spinal cord	D_max_ < 38.54 Gy
Contralateral breast	D_mean_ < 2 Gy
LAD coronary	D_max_ < 17 Gy (if possible)

CTVs, clinical target volumes; IMC, internal mammary chain; CLN, clavicular lymph node; LAD, left anterior descending.

The dose prescription, PTV margins, and dose constraints were derived from the clinical trial Adaptive Radiotherapy in Hypersensitive Patients and High Locoregional Risk Breast Cancer With ETHOS Technology (SAHARA-04) (18).

### Ethos workflow emulation

The Ethos workflow for breast cancer was reproduced using a Varian Ethos emulator (v1.1, Varian Medical Systems, Palo Alto, CA, USA). First, the cone beam computed tomography (CBCT) scan is imported into the emulator, and the synthetic CT (sCT) is designed using DIR to create the deformation matrix. However, during an adaptive couch session, the sCT was not displayed. Only the CBCT scan was visible. As recommended by Varian (Ethos manual), the body and bone contours (resulting from the synthetic CT) shown on the CBCT were verified by the user for each session to ensure the quality of the synthetic CT. Next, the AI generates the contours of the influencer structures (also known as organs that influence the shape and position of the target), namely, the right and left breasts (or chest walls), both lungs, and the heart. The contours of the target volumes are then generated by elastic or rigid registration, according to the user’s choice, to define the IMC and CLNs. The target propagation is based on structure-guided deformations (resulting from the influencer and bone structures generated in the previous step). The CTV_Breast and CTV_Chestwall are derived structures from the breast and chest wall (which are influencer structures) excluding the 5 mm beneath the skin. The CTV_Boost is a derived structure resulting from an expansion around the surgical fiducials (defined as target volume). During the workflow, the volumes created by Ethos were not edited in order to evaluate the performance of the system. Finally, the adapted and scheduled plans are generated. The scheduled plan is the initial plan recalculated on the sCT, while the adapted plan is the initial plan re-optimized and recalculated on the image of the day.

For each patient, five extended CBCT scans performed initially for their treatment were randomly selected in order to simulate five adaptive sessions.

### Contour accuracy

To evaluate the performance of the Ethos in terms of contour accuracy, the generated structures (influencers and target volumes) were compared with the contours manually drawn on the CBCT scans. Two physicians performed and shared the delineations before the study began. The ESTRO consensus guidelines (19, 20) were used to delineate target volumes, breast/wall, and axillary (Berg I); subclavicular (Berg II, III) and supraclavicular (Berg IV) lymph nodes (Nodes hereafter); and IMC. Organs at risk were delineated following French RecoRad 2022 (21) recommendations using TheraPanacea software (22). Structures derived from influencers and target volumes (CTV_Breast/Chestwall and CTV_Boost) were excluded from this part.

Each CBCT was used twice in the emulator: the first time to recover the target volumes by elastic propagation and the second time by rigid propagation. The generated contours by Ethos were evaluated using two metrics: the Dice similarity coefficient (DICE) index and the Hausdorff 95% distance (HD95). These metrics were chosen because of their common use in the literature (23, 24) and their complementary nature. DICE indices were retrieved using Eclipse TPS (v15.6), and HD95 values using 3D Slicer software.

Finally, the repeatability of the AI-generated influencer contours was investigated by repeating the workflow twice and comparing the influencer generation with 3D Slicer.

### Dose assessment

In order to compare the dosimetry of the adapted and scheduled plans for each fraction, several dose–volume histogram parameters were reported. To study the target volumes (boost, breast/chest wall, IMC, and CLN), the dose received by 95% of the volume (D95%) and the homogeneity index were used. The homogeneity index is defined by the following formula (25). For the OARs, the mean dose was recorded for the heart, contralateral breast, and both lungs; the volume receiving 17 Gy (V17Gy) for the ipsilateral lung and the mean dose for the contralateral breast are criteria derived from the dose constraints in the protocol for the clinical trial (18) following this feasibility study (**Table 2**).

The adapted and scheduled plans were also compared with the reference plans, defined as the initial plan performed on the simulation CT.

### Time measurement

Contour generation times by AI, elastic/rigid propagation (without edition), calculation, and optimization were recorded. These times were compared between workflows for patients who had been treated without and with mastectomy (breast and chest wall, respectively). Average times were also calculated for all patients.

### Quality assurance

Ethos includes Mobius3D (version 3.1, Varian Medical Systems), an integrated and independent dose calculation quality assurance tool using the treatment delivery log files. However, our institution’s experience is based on portal dosimetry evaluation. Therefore, quality assurance of the adapted plans was performed using the portal device and the independent software EpiQA (v5.1.3, EPIdos, Bratislava, Slovakia). Each adapted plan was exported to Eclipse TPS, and the planar dose was calculated in a homogeneous cube phantom using the AAA algorithm (Eclipse v15.6, Varian Medical Systems, Palo Alto, CA, USA). Data from the delivered plans were acquired using the portal imager on the Ethos accelerator. The evaluation metric was the global gamma pass rate with the criteria 3%/3mm, 2%/2mm, and 1%/1mm and the field area threshold.

## Results

### Contour accuracy

**Figure 1:**
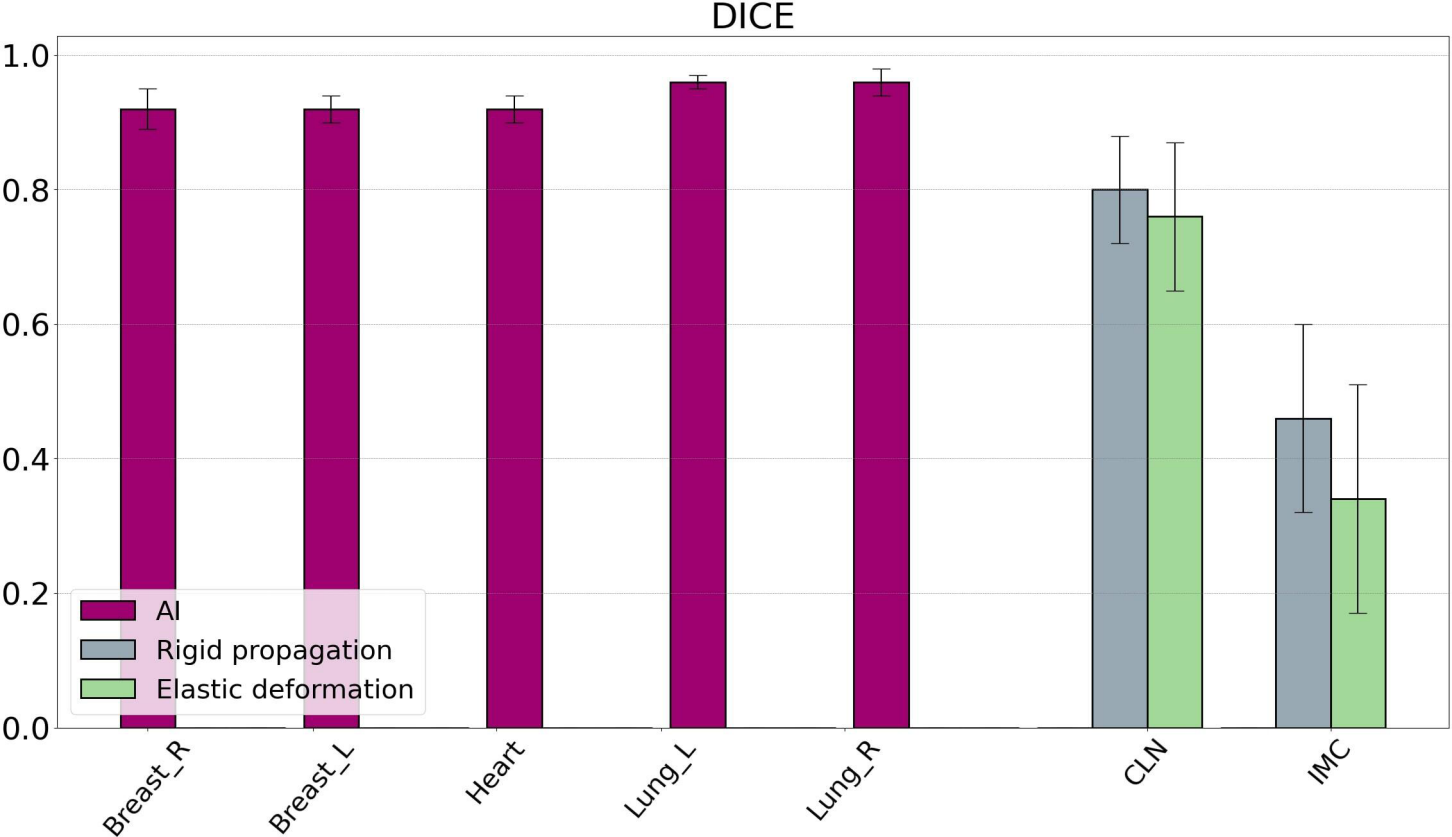
DICE values resulting from the comparison between contours generated by Ethos and contours delineated by physicians. The influencer (from AI generation) DICE results are displayed in purple, while the target volume DICE results are displayed in gray (for rigid propagation) and green (for elastic deformation). Bars are the standard deviations. DICE, Dice similarity coefficient; AI, artificial intelligence.

**Figure 1** shows the DICE values obtained from the comparison between the AI-generated influencer contours and those delineated by the physician. On average, the DICE is always strictly greater than 0.90 and, in the case of the lungs, greater than 0.95. The same applies to the median. The highest value is reached by the right and left lungs, with DICE of 0.96 ± 0.02 and 0.96 ± 0.01, respectively. The lowest is reached by the right breast (0.92 ± 0.03). The right breast has the largest standard deviation, and the right lung has the smallest. Regarding the comparison between rigid and elastic propagations, the average DICE resulting from contours generated by rigid propagation are larger than those for contours generated by elastic propagation: they are 0.8 ± 0.1 and 0.46 ± 0.17 for rigid for the CLN and IMC, respectively, versus 0.76 ± 0.06 and 0.34 ± 0.14 in elastic, again for the CLN and IMC, respectively.

**Figure 2:**
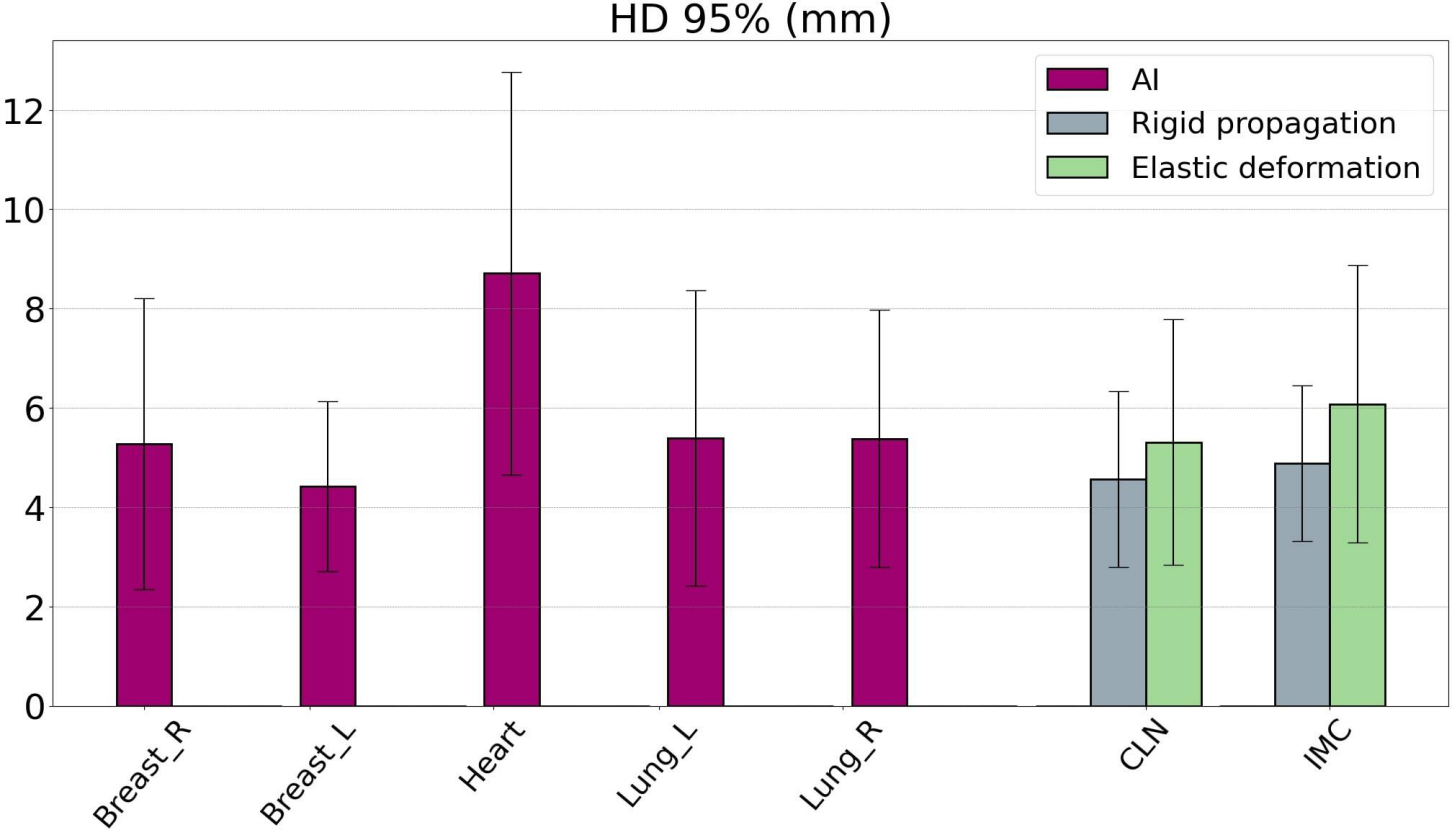
Hausdorff distance 95% (HD95) values resulting from the comparison between contours generated by Ethos and contours delineated by physicians. The influencer (from AI generation) HD95 results are displayed in purple, while the target volume HD95 results are displayed in gray (for rigid propagation) and green (for elastic deformation). AI, artificial intelligence.

**Figure 3:**
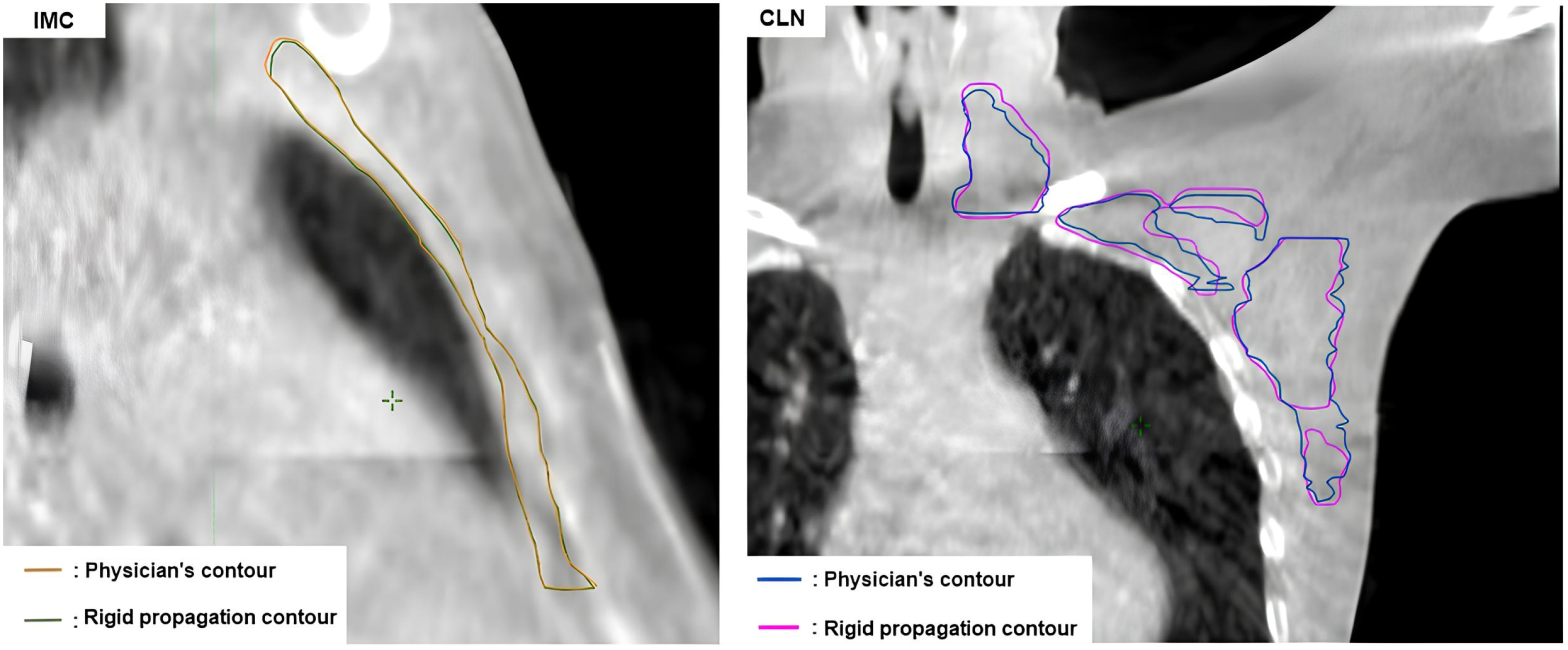
Comparison between the physician contour and the rigid propagation contour for the internal mammary chain (left) and the clavicular lymph nodes (right).

**Figure 2** shows the HD95 values comparing the AI-generated influencer contours with the clinician contours. The mean HD95 does not exceed 10 mm for all influencers. The left breast showed the lowest HD95 and standard deviation with 4.4 ± 1.7 mm. The heart had the highest mean HD95 of 8.8 mm and also the highest variability, with a standard deviation of 4.1 mm. For the right breast and both lungs, HD95 remained between 5 and 6 mm. Similarly, HD95 was lower for rigid contours than for elastic contours in the case of the CLNs: the average was 4.6 ± 1.8 mm in rigid propagation and 5.3 ± 1.6 mm in elastic deformation. For the IMC, the HD95 average was 4.9 ± 2.5 mm for rigid contours and 4.4 ± 2.8 mm for elastic contours. **Figure 3** displays an example of a comparison between the physician contour and the rigid propagation contour for the internal mammary chain (left) and the clavicular lymph nodes (right).

### Repeatability

The results for the repeatability of influencer generation by AI presented in **Table 3** show an average DICE close to 1 and an average HD95 of 0.1 mm.

**Table 3:** Averages of DICE and HD95 indices (in mm) resulting from comparison of contours obtained by AI for influencers.

	DICE	HD95 (mm)
Right breast	1.0	0.1 ± 0.3
Left breast	1.0	0.1 ± 0.3
Heart	1.0	0.1 ± 0.4
Left lung	1.0	0.1 ± 0.3
Right lung	1.0	0.1 ± 0.3

DICE, Dice similarity coefficient; HD95, Hausdorff 95% distance; AI, artificial intelligence.

### Generating time

The workflow can be divided into several stages, each requiring a different amount of time: the time needed to generate contours using AI and propagation and then the time needed to calculate and optimize the treatment plans.

The mean contour generation time per AI was 73 s. Mean target volume contour generation times and associated standard deviations were longer for patients without mastectomy but not significant (85 ± 9 s for breast *vs.* 76 ± 6 s for chest wall). Calculation times averaged between 50 and 55 s, and optimization times were the longest, averaging between 120 and 130 s (**Table 4**). The total time for a complete workflow, from creating the influencer to calculating the dose, was 5 to 6 minutes, not including editing.

**Table 4:** Average contour generation times using AI and propagation, calculation times, and plan optimization times in seconds.

Localizations	Influencer generation time (using AI)	Target volume generation time (using contour propagation)	Dose calculation time	Optimization time	Total (s)
Breast	80 ± 4	85 ± 9	55 ± 3	128 ± 11	348 ± 22
Chest wall	73 ± 4	76 ± 6	52 ± 2	121 ± 6	322 ± 11
Global	73 ± 4	80 ± 9	53 ± 3	125 ± 9	331 ± 20

AI, artificial intelligence.

### Dose assessment

In view of the results obtained in the previous section, it was decided at the mid-study to evaluate only the plans resulting from rigid propagation, as these were of better quality than those resulting from elastic propagation. The dosimetry for adapted and scheduled plans was evaluated for each fraction, and some dose metrics were evaluated based on the constraints. In addition, some metrics resulting from dose constraints (**Table 2**) were excluded from the comparison, such as V17Gy or V35Gy for the heart, because the results were too small and not relevant to the study.

**Table 5** shows that the homogeneity index remains below 0.1 on average for the adapted plans, except for the breast, where the homogeneity index (HI) is 0.17 ± 0.09. It is greater than or equal to 0.1 on average for scheduled plans, regardless of the target. We also noted that the CTV_Breast has the highest HI and standard deviation among target volumes, whether with adapted or scheduled plans.

**Table 5:** Average homogeneity index for target volumes.

HI	Boost	Breast/chest wall	IMC	CLN
Adapted plan	0.07 ± 0.02	0.17 ± 0.09	0.09 ± 0.02	0.07 ± 0.02
Scheduled plan	0.1 ± 0.06	0.21 ± 0.11	0.12 ± 0.05	0.11 ± 0.05

HI, homogeneity index; IMC, internal mammary chain; CLN, clavicular lymph node.

In terms of average dose delivered to target volumes, the adapted plans delivered higher D95% than the scheduled plans (**Table 6**). Furthermore, the D95% for the scheduled plan was inferior to the dose results of the reference plans.

**Table 6:** Average D95% (Gy) delivered to target organs for adapted and scheduled plans.

D95% (Gy)	Boost	Breast/chest wall	IMC	CLN
Adapted plan	49.91 ± 0.24	40.97 ± 0.4	40.21 ± 0.37	41.05 ± 0.26
Scheduled plan	48.88 ± 1.52	40.28 ± 1.09	39.29 ± 1.71	40.25 ± 1.51
Reference plan	49.83 ± 0.36	40.82 ± 0.36	40.16 ± 0.43	40.81 ± 0.37

D95%, dose received by 95% of the volume; IMC, internal mammary chain; CLN, clavicular lymph node.

**Figure 4:**
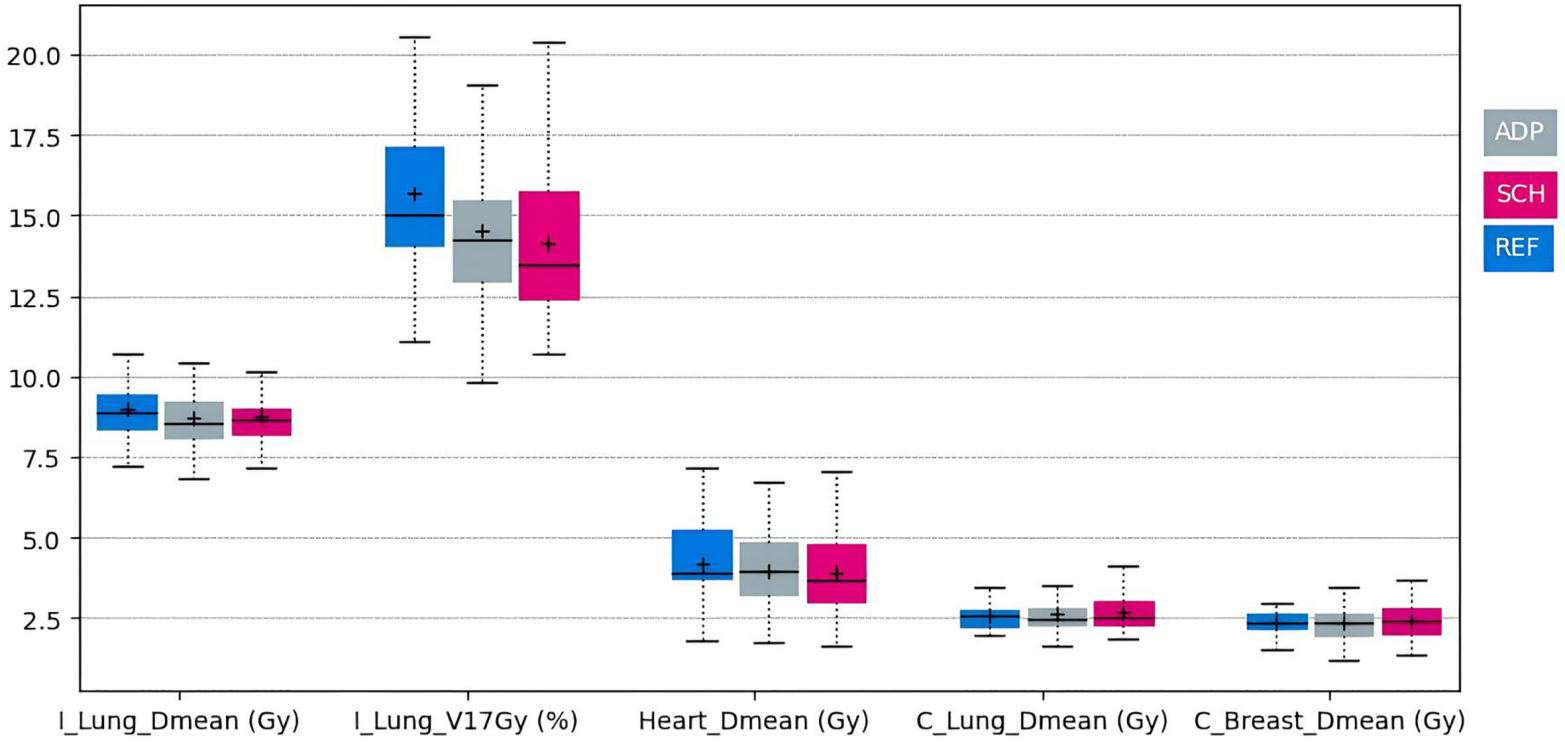
Box plots of doses received by the heart, the ipsilateral lung (I_Lung), and the contralateral lung (C_lung) for reference, adapted, and scheduled plans (blue, gray, and pink, respectively).

The average dose received by the OARs was similar between the adapted and scheduled plans, in terms of both mean dose received and standard deviations (**Figure 4**). The largest difference between adapted and scheduled plans was found in the ipsilateral lung location, with a mean dose difference of 0.09 Gy. The same results between adapted and scheduled plans were found for specific organs, as reflected by the median: 8.6 Gy for the ipsilateral lung, 2.5 Gy for the contralateral lung, and 4 Gy for the heart. Furthermore, the dose results obtained for both adapted and scheduled plans were equivalent or inferior to those of the reference plans.

### Gamma index

**Figure 5:**
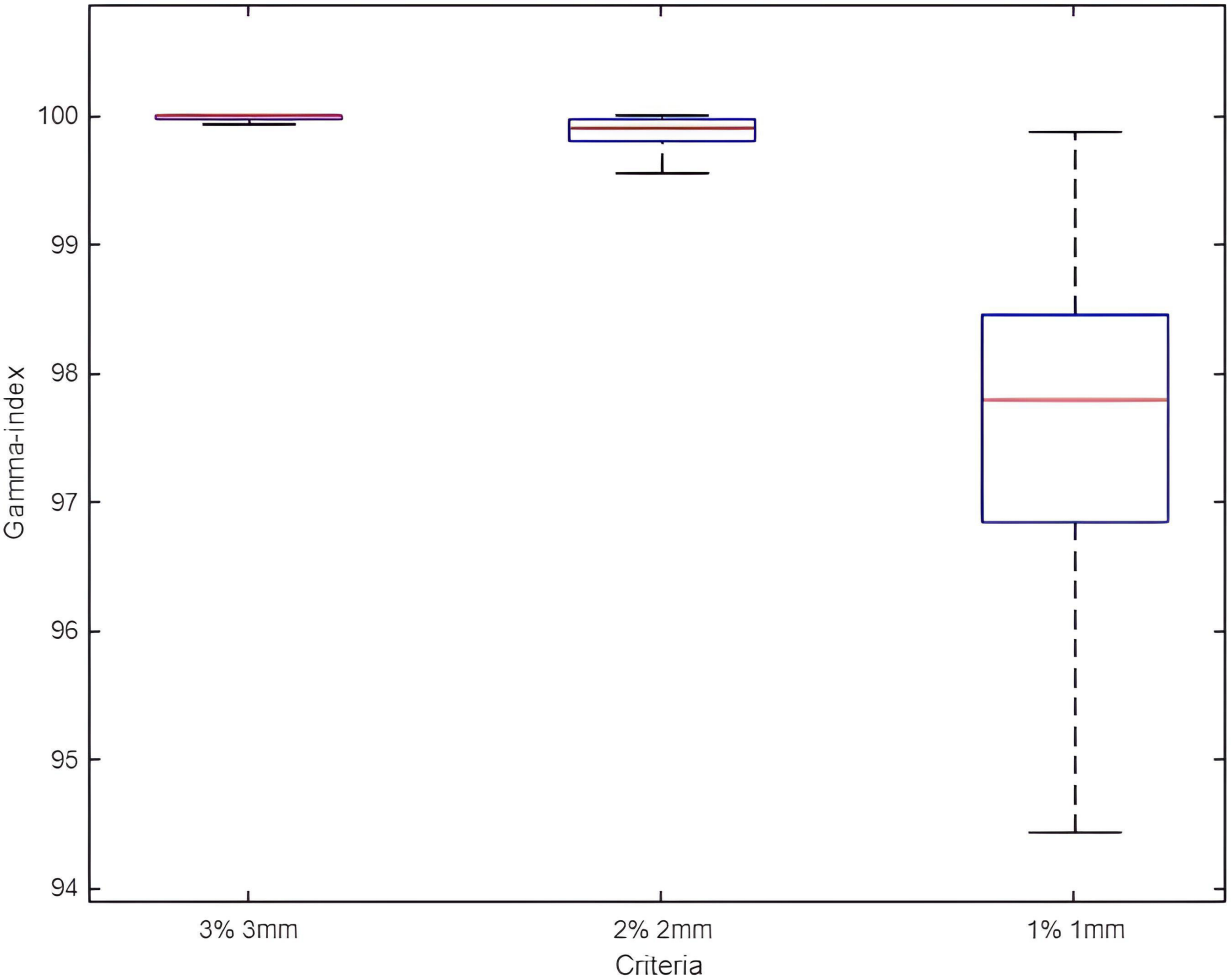
Box plots of gamma index pass rate results for all adapted plans with 3%/3mm, 2%/2mm, and 1%/1mm criteria.

Regardless of the parameter used, the mean gamma index pass rates remained above 95%. For a criterion of 3%/3mm, the gamma index was 99.93% ± 0.38%. The standard deviation was the highest for the 1%/1mm criterion (**Figure 5**).

## Discussion

The aim of this study was to evaluate the feasibility of adaptive radiotherapy with Ethos for breast cancer by assessing the different steps: accuracy of contour generation, quality of the scheduled and adapted plan, and quality assurance of treatment delivery.

From the results discussed above, the influencer contours generated by AI are consistent and reproducible with those delineated by the physician. To assess the consistency of our results, we compared our results with two studies using the same metrics. The first is a study by S. S. Almberg et al. (26), which aimed to “train and evaluate a deep-learning contouring model for locoregional breast cancer for clinical implementation”. This comparison shows the consistency of our results: the right breast and CLNs are equivalent in their values. Lung and left breast results are similar for DICE, but there is an approximate 4-mm difference for HD95. The second study, by N. Baks et al. (27), compared the results of two datasets resulting from a deep-learning-based segmentation model for patients with locoregional breast cancer. A higher DICE and lower HD95 indicate a better correlation with the physician contours. Comparing our results with those of the study, we also noted the consistency of our results: whether we considered DICE or HD95, the results were very similar. We found the same DICE averages for the right and left breasts, heart, and both lungs. However, for the HD95, the averages differ by 3.6 mm for the right breast. The differences observed may be explained by the data used since the study by S. S. Almberg et al. used CT data from 200 patients with left breast cancer only, whereas the study by N. Baks et al. used data from 30 patients, equally divided between right and left breast cancer. As a reminder, we used five CBCT scans per patient, with 20 patients equally divided between right/left breast and right/left wall. In addition, Almberg et al. and N. Baks et al. generated all their contours by AI, while the contours of our target volumes (IMC and CLN) were obtained by propagation.

Our results are more contrasted for target volume contours generated by elastic or rigid propagation. It can be seen that contours generated by elastic deformation are less satisfactory than those generated by rigid propagation. In terms of DICE, the mean and median are higher for rigid than for elastic deformation, regardless of whether we considered the CLN or IMC. Similarly, for HD95, the median and mean are lower for rigid deformation than for elastic deformation for the CLN, and the median only follows the same trend for the IMC.

However, these results must be qualified: whether in elastic or rigid propagation, the target volume contours generated by Ethos are not usable, as they stand without revision. Indeed, even in the case of rigid propagation, which seems to offer better results than elastic deformation, the average DICE of the IMC does not exceed 50%, and that of the CLN does not exceed 80%, which remains insufficient for clinical use.

However, the HD95 of the influencer and target volume contours remain within a range of 4 to 6 mm (except for the heart). This raises questions about the limitations of DICE, which offers a higher index for larger organs. The IMC is a good illustration of these limitations since it is the smallest volume and consequently has the lowest DICE, while its HD95 values are in the same range as those of the other sites.

Although the time taken to generate contours and treatment plans is reasonable for clinical use, it is longer than for conventional treatment. This is because the clinician has to revise some of the contours (particularly the target volumes), which increases the treatment time. As a result, adaptive radiotherapy with Ethos for breast cancer will only benefit a limited number of patients due to the additional workload (time and human resources).

Looking at the dosimetry results, the adapted plans provided better target coverage than the scheduled plans with equivalent OAR protection. However, ART-generated contours have smaller margins than conventional radiotherapy due to daily recontouring and replanning. Thus, despite this equivalence in OAR protection, we can presume that ART treatment using smaller margins than a conventional treatment certainly decreases the dose to the healthy tissue and could reduce toxicity in our case (28). Several studies have already shown that PTV margin reduction can further improve clinical outcomes for accelerated partial breast irradiation (APBI) (29, 30).

Quality assurance using EpiQA demonstrated that the Ethos accelerator was able to deliver adapted plans with acceptable gamma pass rates of 99.93% ± 0.38% for a criterion of 3%/3mm.

To address the limitations of this study, we can mention its mathematical nature. In fact, our data and results come from calculation-based metrics. We chose not to have clinicians modify any of the contours in this feasibility study in order to evaluate the inherent performance of the Ethos solution. However, to verify the use of Ethos in clinical implementation, whether contours (and edition time) or dosimetry, a medical assessment will be necessary and will be the subject of a future study. Similarly, we compared our Ethos contours with contours manually created by physicians: the latter is not exactly repeatable and may be biased (31, 32).

## Conclusion

This study comprehensively evaluated the Ethos adaptive workflow for breast cancer and its potential technical limitations. Although the results demonstrated the high accuracy of AI segmentation and the superiority of adapted plans in terms of target volume coverage, a medical assessment is still required.

## Data availability statement

The raw data supporting the conclusions of this article will be made available by the authors, without undue reservation.

## Ethics statement

Ethical approval was not required for the study involving humans in accordance with the local legislation and institutional requirements. Written informed consent to participate in this study was not required from the participants or the participants’ legal guardians/next of kin in accordance with the national legislation and the institutional requirements.

## Author contributions

AG: Conceptualization, Data curation, Investigation, Writing – original draft. JP: Conceptualization, Formal Analysis, Investigation, Methodology, Supervision, Validation, Writing – review & editing. NM: Data curation, Writing – review & editing. AM: Validation, Writing – review & editing. CB: Validation, Writing – review & editing. NA: Validation, Writing – review & editing. DA: Validation, Writing – review & editing. PF: Supervision, Validation, Writing – review & editing.
